# Diabetes Care Barriers, Use, and Health Outcomes in Younger Adults With Type 1 and Type 2 Diabetes

**DOI:** 10.1001/jamanetworkopen.2023.12147

**Published:** 2023-05-05

**Authors:** Catherine Pihoker, Barbara H. Braffett, Thomas J. Songer, William H. Herman, Melinda Tung, Shihchen Kuo, Anna Bellatorre, Elvira Isganaitis, Elizabeth T. Jensen, Jasmin Divers, Ping Zhang, David M. Nathan, Kimberly Drews, Dana Dabelea, Philip S. Zeitler, Writing Committee for the SEARCH for Diabetes in Youth Study Group and the TODAY Study Group

**Affiliations:** 1 Department of Pediatrics, University of Washington, Seattle; 2 The Biostatistics Center, George Washington University, Rockville, Maryland; 3 Department of Epidemiology, Graduate School of Public Health, University of Pittsburgh, Pittsburgh, Pennsylvania; 4 Departments of Internal Medicine and Epidemiology, University of Michigan, Ann Arbor; 5 Lifecourse Epidemiology of Adiposity and Diabetes Center, University of Colorado Anschutz Medical Campus, Aurora; 6 Research Division, Joslin Diabetes Center and Department of Pediatrics, Harvard Medical School, Boston, Massachusetts; 7 Department of Epidemiology and Prevention, Wake Forest School of Medicine, Winston-Salem, North Carolina; 8 Division of Health Services Research, New York University Long Island School of Medicine, Mineola; 9 Division of Diabetes Translation, Centers for Disease Control and Prevention, Atlanta, Georgia; 10 Diabetes Center Massachusetts General Hospital, Harvard Medical School, Boston; 11 University of Colorado School of Medicine, Aurora

## Abstract

**Importance:**

Treatment challenges exist for younger adults with type 1 (T1D) and type 2 diabetes (T2D). Health care coverage, access to, and use of diabetes care are not well delineated in these high-risk populations.

**Objective:**

To compare patterns of health care coverage, access to, and use of diabetes care and determine their associations with glycemia among younger adults with T1D and with T2D.

**Design, Setting, and Participants:**

This cohort study analyzed data from a survey that was jointly developed by 2 large, national cohort studies: the SEARCH for Diabetes in Youth (SEARCH) study, an observational study of individuals with youth-onset T1D or T2D, and the Treatment Options for Type 2 Diabetes in Adolescents and Youth (TODAY) study, a randomized clinical trial (2004-2011) followed by an observational study (2012-2020). The interviewer-directed survey was administered during in-person study visits in both studies between 2017 and 2019. Data analyses were performed between May 2021 and October 2022.

**Main Outcomes and Measures:**

Survey questions addressed health care coverage, usual sources of diabetes care, and frequency of care use. Glycated hemoglobin (HbA_1c_) levels were assayed in a central laboratory. Patterns of health care factors and HbA_1c_ levels were compared by diabetes type.

**Results:**

The analysis included 1371 participants (mean [range] age, 25 [18-36] years; 824 females [60.1%]), of whom 661 had T1D and 250 had T2D from the SEARCH study and 460 had T2D from the TODAY study. Participants had a mean (SD) diabetes duration of 11.8 (2.8) years. More participants with T1D than T2D in both the SEARCH and TODAY studies reported health care coverage (94.7%, 81.6%, and 86.7%), access to diabetes care (94.7%, 78.1%, and 73.4%), and use of diabetes care (88.1%, 80.5%, and 73.6%). Not having health care coverage was associated with significantly higher mean (SE) HbA_1c_ levels in participants with T1D in the SEARCH study (no coverage, 10.8% [0.5%]; public, 9.4% [0.2%]; private, 8.7% [0.1%]; *P* < .001) and participants with T2D from the TODAY study (no coverage, 9.9% [0.3%]; public, 8.7% [0.2%]; private, 8.7% [0.2%]; *P* = .004). Medicaid expansion vs without expansion was associated with more health care coverage (participants with T1D: 95.8% vs 90.2%; participants with T2D in SEARCH: 86.1% vs 73.9%; participants with T2D in TODAY: 93.6% vs 74.2%) and lower HbA_1c_ levels (participants with T1D: 9.2% vs 9.7%; participants with T2D in SEARCH: 8.4% vs 9.3%; participants with T2D in TODAY: 8.7% vs 9.3%). The T1D group incurred higher median (IQR) monthly out-of-pocket expenses than the T2D group ($74.50 [$10.00-$309.00] vs $10.00 [$0-$74.50]).

**Conclusions and Relevance:**

Results of this study suggested that lack of health care coverage and of an established source of diabetes care were associated with significantly higher HbA_1c_ levels for participants with T1D, but inconsistent results were found for participants with T2D. Increased access to diabetes care (eg, through Medicaid expansion) may be associated with improved health outcomes, but additional strategies are needed, particularly for individuals with T2D.

## Key Points

**Question:**

How do health care coverage, access to, and use of diabetes care vary by diabetes type in younger adults, and what are their associations with glycemic control, as assessed by glycated hemoglobin (HbA_1c_) levels?

**Findings:**

In this cohort study of 1371 participants in 2 large, national cohorts, less health care coverage, access to, and use of diabetes care were reported in individuals with type 2 diabetes compared with those with type 1 diabetes. Less access was associated with higher HbA_1c_ levels, and state-level Medicaid expansion after the Affordable Care Act was associated with higher frequencies of health care coverage and lower HbA_1c_ levels.

**Meaning:**

Findings of this study suggest that increased access to diabetes care, such as through Medicaid expansion, is associated with improved health outcomes in younger adults with diabetes.

## Teaser

This cohort study examines health, socioeconomic, structural, and other factors associated with access to and use of diabetes care in younger adults.

## Introduction

The incidence and prevalence of both youth-onset type 1 diabetes (T1D) and type 2 diabetes (T2D) are increasing.^1,2^ Lower levels of glycemia, as measured by glycated hemoglobin (HbA_1c_), are well established to reduce the risk of long-term complications in both T1D and T2D.^3,4^ Attaining desired HbA_1c_ levels involves daily self-care activities by the individual with diabetes and management by a clinical team.^4^

Several barriers may impede optimal care for individuals with diabetes aged 18 to 35 years. While some of these barriers are the same for patients with diabetes of all ages, the psychosocial demands and transitions unique to younger adults may magnify the implications of some of these barriers. Identified barriers include health care costs, limited access to diabetes care, and social and structural factors that limit adoption of best care practices.^5,6^ Approximately one-half of youth with T1D reported high health care costs as a barrier to routine care; youths in households with low income and those without health care coverage were most likely to report such a barrier.^7^ In a national sample of privately insured patients with T1D, the mean annual out-of-pocket expenses (OOPEs) were $2298 for children with T1D, with 8% of the sample reporting annual OOPEs exceeding $5000.^8^ The SEARCH for Diabetes in Youth (SEARCH) study found that 60% of adolescents and young adults with T1D had OOPEs of at least $50 per month.^5^ There are limited data on OOPEs in youth and young adults with diabetes, particularly T2D.

Young adults with diabetes face unique challenges during the transition from pediatric to adult diabetes care.^9^ Health care coverage may be limited, interrupted, or absent for younger adults who turn age 26 years and are no longer eligible to remain on their families’ health insurance plans. Interrupted access to care during this period can affect health care quality, costs, and outcomes.^9,10,11^ Young adults with T1D who reported lack of health care coverage had 3.5-fold greater odds of a prolonged gap between pediatric and adult diabetes care.^12^ Young adults with T2D who lacked health care coverage were less likely to have ambulatory diabetes care and more likely to have higher HbA_1c_ levels.^13^

The extent to which barriers to care exist for youth and younger adults with diabetes and the factors associated with them are not fully understood. Furthermore, how the constellation of health care access, use, and OOPEs are associated with HbA_1c_ level in T1D and T2D remains largely unreported. In this cohort study, we aimed to compare patterns of health care coverage, access to, and use of diabetes care and assessed their associations with glycemia among younger adults with T1D and those with T2D enrolled in the SEARCH and Treatment Options for Type 2 Diabetes in Adolescents and Youth (TODAY) studies.

## Methods

All participants or their parents or guardians provided written informed consent or assent as appropriate for each participant’s age and according to local guidelines. Twenty-eight Institutional Review Boards approved the present cohort study (7 for the SEARCH study and 21 for the TODAY study; eAppendix in Supplement 1). We followed the Strengthening the Reporting of Observational Studies in Epidemiology (STROBE) reporting guideline.

The SEARCH study cohort was recruited from the population-based SEARCH Registry, which since 2002 has continuously ascertained youth-onset T1D and T2D cases from Colorado, selected Southwestern American Indian reservations in Arizona and New Mexico, Ohio, Washington, South Carolina, and California.^14^ Between 2002 and 2015, participants who were diagnosed with T1D or T2D were enrolled in the SEARCH study shortly after diagnosis (baseline visit at a mean diabetes duration of 10 months), with 3549 participants (3017 with T1D and 532 with T2D) eligible for a follow-up visit. Data for the present study were collected for 661 participants with T1D and 250 participants with T2D at in-person study visits between January 27, 2017, and September 30, 2019. All participants included in this study were 18 years or older. Compared with participants who were not included in the present analyses, those with T1D were similar in the distribution of diabetes duration and body mass index (BMI) *z* score at baseline, while those with T2D were similar in the distribution of diabetes duration, sex, race and ethnicity, parental educational level, BMI, and HbA_1c_ level at baseline. Participants with T1D were older, had a lower HbA_1c_ level, were less likely to be male and non-Hispanic White individuals, had parents with a lower educational level, and had a larger waist circumference compared with nonparticipants with T1D, and participants with T2D were older and had a larger waist circumference than nonparticipants with T2D.

As previously described,^15^ the TODAY study enrolled 699 participants, who were aged 10 to 17 years and had a T2D diagnosis of less than 2 years’ duration, in a randomized clinical trial between 2004 and 2009, and followed them through 2011. From 2012 to 2020, 518 participants were enrolled in the observational follow-up phase of the study, with annual visits and medical management provided by their diabetes care practitioners. Data for the present study were obtained from 460 participants (aged ≥18 years) who completed annual visits between April 1, 2017, and May 7, 2019. For participants who completed more than 1 visit in this time frame, data from their most recent visit were used. Compared with the TODAY study participants who were not included in the present analyses (n = 239), the cohort was similar in the distribution of sex, race and ethnicity, household educational level, diabetes duration, BMI, waist circumference, and HbA_1c_ level at entry into the TODAY study. Participants included in the current study were slightly younger than those who were not.

In both the SEARCH and TODAY studies, demographic data, medical history, and current medications were self-reported, and HbA_1c_ measurements were performed centrally at the Northwest Lipid Metabolism and Diabetes Research Laboratories at the University of Washington, Seattle, Washington.^16^ Participants self-reported their household income and educational levels as well as race and ethnicity using the following categories selected from the SEARCH and TODAY studies: American Indian, Asian or Pacific Islander, Hispanic, non-Hispanic Black, or non-Hispanic White. The level of depressed mood was measured using the Center for Epidemiologic Studies Depression Scale (CES-D) in the SEARCH study and by the 8-item Patient Health Questionnaire (PHQ-8) in the TODAY study. A CES-D total score of 16 or higher or a PHQ-8 total score of 5 or higher indicates depressed mood.^17^

The SEARCH and TODAY studies jointly developed an interviewer-administered survey to collect economic data in each study cohort (eTable 1 in Supplement 1). The survey was administered at in-person visits in both studies between 2017 and 2019. Questions in the survey addressed having an established diabetes care practitioner (usual source of care for diabetes), frequency of visits for diabetes care in the previous 6 months, barriers to access, health care coverage in the previous year, and OOPEs. Wording and structure of the questions were adapted from questions in the Household Component of the Medical Expenditure Panel Survey.^18,19^ In the present study, the term health care coverage refers to commercial health insurance; public health insurance, such as Medicaid; or access to services through the Indian Health Service or Tribal Nations. Health care coverage status and type were collapsed into: no, public (Medicaid, Medicare, Children’s Health Insurance Program, military [TRICARE or Civilian Health and Medical Program of the Uniformed Services], Indian Health Service or Tribal Nations, or other state or federal), and private (through work, parent or caregiver, or purchased individually).

With the implementation of the Affordable Care Act, many states expanded health care coverage to eligible residents through Medicaid expansion. Participants in both studies were classified as residents of states with Medicaid expansion or without Medicaid expansion at the time of survey administration^20^ (eFigure in Supplement 1).

### Statistical Analysis

Results are presented separately by study cohort and diabetes type (T1D and T2D in SEARCH, and T2D in TODAY). Since the SEARCH study was an observational study from inception and the TODAY study started as an intervention study (2004-2011), with participants meeting run-in criteria prior to enrollment, the study teams elected to separately analyze results from participants with T2D in the SEARCH and TODAY studies. Thus, the results were compared only between the participants with T1D and T2D from the SEARCH study. We also examined whether patterns observed in the population with T2D in the SEARCH study were supported in the larger the TODAY study sample of participants with T2D.

Mean OOPEs were calculated as the weighted mean based on the midpoint for each interval and the number of participants whose income fell within each interval. The midpoint of the last interval was estimated at $309 based on 2020 MarketScan data. Linear regression models were used to evaluate the association between HbA_1c_ level and health care coverage, usual source of care, and health care use groups. Adjustments were made for age, diabetes duration, educational level, and depressed mood. Models for participants with T1D were further adjusted for insulin pump use and frequency of glucose monitoring. Models for participants with T2D were further adjusted for diabetes medications.

All analyses were performed using SAS, version 9.4 (SAS Institute Inc). A 2-sided *P* < .05 was considered statistically significant. Data analyses were performed between May 2021 and October 2022.

## Results

The present study included 1371 participants, with a mean (range) age of 25 (18-36) years, 824 females (60.1%) and 547 males (39.9%), and a mean (SD) duration of diabetes of 11.8 (2.8) years. Characteristics of both study cohorts at the time of the survey (2017 to 2019) are presented in Table 1. The SEARCH study participants with T1D (n = 661) were slightly younger at diabetes diagnosis compared with participants with T2D in the SEARCH (n = 250) and TODAY (n = 460) studies. There was a higher proportion of participants with T2D who identified as non-Hispanic Black or Hispanic compared with those with T1D. Treatment data showed that over 50% of participants with T2D used insulin. Glycemic control was suboptimal, with mean HbA_1c_ levels that were substantially higher than the recommended targets among all groups.

**Table 1.** Demographic and Clinical Characteristics of the SEARCH and TODAY Study Cohorts at Survey Time^a^

Characteristic	SEARCH study cohort, No. (%)		TODAY T2D study cohort, No. (%)
	T1D	T2D	
All participants	661 (100)	250 (100)	460 (100)
Age at time of survey, mean (SD), y	23.4 (3.8)	24.8 (3.9)	26.2 (2.4)
Age at time of survey, median (range), y	22.8 (18.0-36.0)	24.3 (18.2-35.6)	26.1 (19.2-32.1)
≥26 y of age at time of survey	160 (24.2)	82 (32.8)	240 (52.2)
Age at diagnosis, mean (SD), y	11.4 (3.8)	14.6 (2.5)	13.6 (2.1)
Sex			
Female	363 (54.9)	161 (64.4)	300 (65.2)
Male	298 (45.1)	89 (35.6)	160 (34.8)
Race and ethnicity^b^			
Hispanic	148 (22.4)	65 (26.0)	179 (38.9)
Non-Hispanic Black	118 (17.9)	118 (47.2)	161 (35.0)
Non-Hispanic White	370 (56.0)	49 (19.6)	87 (18.9)
Other^c^	25 (3.8)	18 (7.2)	33 (7.2)
Educational level			
<High school	52 (7.9)	31(12.4)	46 (10.0)
High school, GED, or business or technical school diploma	193 (29.2)	96 (38.4)	303 (65.9)
<College	289 (43.7)	101 (40.4)	47 (10.2)
College or graduate degree	118 (17.9)	20 (8.0)	64 (13.9)
Unknown	9 (1.4)	2 (0.8)	NA
Annual household income, $			
<25 000	363 (54.9)	126 (50.4)	268 (62.9)
25 000-49 999	91 (13.8)	27 (10.8)	123 (28.9)
≥50 000	60 (9.1)	2 (0.8)	35 (8.2)
Don’t know or refused to answer	147 (22.2)	95 (38.0)	0
Employment status			
Full-time	258 (39.0)	82 (32.8)	267 (58.0)
Part-time	201 (30.4)	59 (23.6)	82 (17.8)
Unemployed	99 (15.0)	55 (22.0)	88 (19.1)
Student	53 (8.0)	11 (4.4)	11 (2.4)
Disabled	18 (2.7)	28 (11.2)	12 (2.6)
Unknown	32 (4.8)	15 (6.0)	0
Household members, median (range)	3 (1-11)	4 (1-12)	3 (1-12)
Depressed mood^d^	177 (26.9)	90 (36.0)	138 (30.2)
Diabetes medications			
Metformin only	1 (0.2)	30 (12.8)	91 (19.8)
Insulin only or with other medications	646 (98.5)	135 (57.5)	237 (51.5)
Other or none	9 (1.4)	70 (29.8)	132 (28.7)
Insulin pump use	313 (48.9)	NA	NA
Frequency of glucose monitoring, ≥3 per d	424 (68.0)	NA	NA
Continuous glucose monitoring use	200 (30.6)	NA	NA
Duration of diabetes, mean (SD), y	12.0 (3.3)	10.1 (3.6)	12.5 (1.5)
HbA_1c_ level, mean (SD), %	9.0 (2.1)	9.5 (2.9)	9.3 (2.8)
HbA_1c_ level, mean (SD), mmol/mol	74.7 (23.4)	80.5 (31.6)	78.3 (30.3)

Abbreviations: GED, General Educational Development; HbA_1c_, glycated hemoglobin; NA, not available; SEARCH, SEARCH for Diabetes in Youth; T1D, type 1 diabetes; T2D, type 2 diabetes; TODAY, Treatment Options for Type 2 Diabetes in Adolescents and Youth.

^a^ These analyses included SEARCH study visits conducted between January 27, 2017, and September 30, 2019, and TODAY study visits conducted between April 1, 2017, and May 7, 2019. Two participants at the Colorado site who were part of both SEARCH and TODAY studies were included only in the SEARCH sample since they had enrolled in the SEARCH study prior to enrolling in the TODAY study.

^b^ Race and ethnicity were self-reported by participants in the SEARCH and TODAY studies.

^c^ Other race and ethnicity included American Indian and Asian or Pacific Islander.

^d^ Depressed mood was assessed by the Center for Epidemiologic Studies Depression Scale in the SEARCH study and by the 8-item Patient Health Questionnaire in the TODAY study. A Center for Epidemiologic Studies Depression Scale total score of 16 or higher or an 8-item Patient Health Questionnaire total score of 5 or higher indicates depressed mood.

More participants with T1D than T2D in both the SEARCH and TODAY studies reported health care coverage (94.7%, 81.6%, and 86.7%, respectively). Among participants with coverage, most with T1D had private coverage (69.8%), while a higher proportion of participants with T2D reported having public coverage (Table 2). This higher frequency of public coverage reflected the higher proportion of participants with T2D reporting low household income levels. Health care coverage was higher in states with vs without Medicaid expansion among participants with T1D (95.8% vs 90.2%) and participants with T2D in the SEARCH study (86.1% vs 73.9%) and the TODAY study (93.6% vs 74.2%) (eTable 2 in Supplement 1). The proportion of participants in both studies with health care coverage was similar between participants younger than 26 years vs 26 years or older (eTable 3 in Supplement 1).

**Table 2.** Health Care Coverage by Study Cohort

Coverage	SEARCH study cohort, No. (%)		TODAY T2D study cohort, No. (%)
	T1D	T2D	
Health care coverage status in past 12 mo, all participants	661 (100)	250 (100)	460 (100)
No	16 (2.4)	34 (13.6)	61 (13.3)
Yes	626 (94.7)	204 (81.6)	399 (86.7)
Don’t know, refused to answer, or missing data	19 (2.9)	12 (4.8)	0
Health care coverage type, all participants	626 (94.7)	204 (81.6)	399 (86.7)
Public			
Medicaid, Medicare, CHIP, or other state or federal	160 (25.6)	103 (50.5)	182 (45.6)
Military (eg, TRICARE, CHAMPUS)	7 (1.1)	2 (1.0)	0
Indian Health Service or Tribal Nations	0	1 (0.5)	13 (3.3)
Private through work, parent or caregiver, or purchased individually	423 (67.6)	84 (41.2)	177 (44.4)
Mixed public and private	25 (4.0)	8 (3.9)	20 (5.0)
Other type or unknown	11 (1.8)	6 (2.9)	7 (1.8)
Health care coverage status and type, collapsed sample^a^	606 (91.7)	224 (89.6)	433 (94.1)
No coverage	16 (2.6)	34 (15.2)	61 (14.1)
Public	167 (27.6)	106 (47.3)	195 (45.0)
Private	423 (69.8)	84 (37.5)	177 (40.9)

Abbreviations: CHAMPUS, Civilian Health and Medical Program of the Uniformed Services; CHIP, Children’s Health Insurance Program; SEARCH, SEARCH for Diabetes in Youth; T1D, type 1 diabetes; T2D, type 2 diabetes; TODAY, Treatment Options for Type 2 Diabetes in Adolescents and Youth.

^a^ The collapsed sample excluded 31 SEARCH study participants who did not know, refused to answer, or were missing health care status; 33 SEARCH and 20 TODAY study participants with mixed public and private coverage; and 17 SEARCH and 7 TODAY study participants with other or unknown health plan types.

Most participants reported having an established source of diabetes care; however, those with T1D were more likely than those with T2D in both SEARCH and TODAY studies to report having a usual source of care (94.7% 78.1% and 73.4%, respectively) (Table 3). In all 3 groups, participants without health care coverage were far less likely to have an established source of diabetes care than those with coverage. In participants with T1D, the reasons reported for not having an established source of diabetes care included not having health care coverage (30.6%), recent change in health insurance plan (11.1%), unaffordability of care (11.1%), and a recent move (11.1%). In participants with T2D in the SEARCH and TODAY studies, the most common reasons were not having health care coverage (21.3% and 30.7%), seldom or never getting sick (14.9% and 26.3%), not knowing where to go for care (19.2% and 7.9%), and unaffordability of care (8.5% and 5.3%).

**Table 3.** Usual Source of Diabetes Care, Frequency of Use, and OOPEs by Study Cohort and Health Care Coverage Type^a^

	SEARCH study cohort, No. (%)								TODAY T2D study cohort, No. (%)			
	T1D				T2D				All	No coverage	Public coverage	Private coverage
	All	No coverage	Public coverage	Private coverage	All	No coverage	Public coverage	Private coverage				
Usual source of care for diabetes	606 (100)	16 (2.6)	167 (27.6)	423 (69.8)	224 (100)	34 (15.2)	106 (47.3)	84 (37.5)	433 (100)	61 (14.1)	195 (45.0)	177 (40.9)
No	32 (5.3)	8 (50.0)	10 (6.0)	14 (3.3)	48 (21.4)	17 (50.0)	16 (15.1)	15 (17.9)	114 (26.3)	41 (67.2)	34 (17.4)	39 (22.0)
Yes	574 (94.7)	8 (50.0)	157 (94.0)	409 (96.7)	175 (78.1)	16 (47.1)	90 (84.9)	69 (82.1)	318 (73.4)	20 (32.8)	161 (82.6)	137 (77.4)
Don’t know, refused to answer, or missing data	0	0	0	0	1 (0.5)	1 (2.9)	0	0	1 (0.2)	0	0	1 (0.6)
Frequency of use in past 6 mo	n = 574	n = 8	n = 157	n = 409	n = 175	n = 16	n = 89	n = 69	n = 318	n = 20	n = 161	n = 137
0	69 (12.0)	3 (37.5)	15 (9.6)	51 (12.5)	34 (19.4)	1 (6.3)	13 (14.6)	20 (29.0)	84 (26.4)	9 (45.0)	37 (23.0)	38 (27.7)
1	203 (35.4)	1 (12.5)	52 (33.1)	150 (36.7)	61 (35.1)	9 (56.3)	26 (29.2)	26 (37.7)	104 (32.7)	6 (30.0)	48 (29.8)	50 (36.5)
2	246 (42.9)	3 (37.5)	65 (41.4)	178 (43.5)	47 (27.0)	3 (18.8)	32 (36.0)	12 (17.4)	85 (26.7)	3 (15.0)	48 (29.8)	34 (24.8)
≥3	56 (9.8)	1 (12.5)	25 (15.9)	30 (7.3)	32 (18.4)	3 (18.8)	18 (20.2)	11 (15.9)	45 (14.2)	2 (10.0)	28 (17.4)	15 (11.0)
Don’t know, refused to answer, or missing data	0	0	0	0	0	0	0	0	0	0	0	0
Mean OOPEs per mo, $	n = 606	n = 16	n = 167	n = 423	n = 224	n = 34	n = 106	n = 84	n = 433	n = 61	n = 195	n = 177
0	85 (14.0)	2 (12.5)	67 (40.1)	16 (3.8)	85 (38.0)	15 (44.1)	58 (54.7)	12 (14.3)	171 (39.5)	37 (60.7)	109 (55.9)	25 (14.1)
1-19	51 (8.4)	0	30 (18.0)	21 (5.0)	21 (9.4)	0	17 (16.0)	4 (4.8)	58 (13.4)	2 (3.3)	39 (20.0)	17 (9.6)
20-49	47 (7.8)	1 (6.3)	14 (8.4)	32 (7.6)	32 (14.3)	1 (2.9)	10 (9.4)	21 (25.0)	65 (15.0)	7 (11.5)	16 (8.2)	42 (23.7)
50-99	93 (15.4)	7 (43.8)	15 (9.0)	71 (16.8)	19 (8.5)	3 (8.8)	7 (6.6)	9 (10.7)	46 (10.6)	3 (4.9)	6 (3.1)	37 (20.9)
100-199	104 (17.2)	2 (12.5)	11 (6.6)	91 (21.5)	13 (5.8)	2 (5.9)	3 (2.8)	8 (9.5)	41 (9.5)	8 (13.1)	6 (3.1)	27 (15.3)
≥200	132 (21.8)	3 (18.8)	15 (9.0)	114 (27.0)	28 (12.5)	9 (26.5)	2 (1.9)	17 (20.2)	27 (6.2)	3 (4.9)	3 (1.5)	21 (11.9)
Don’t know, refused to answer, or missing data	94 (15.5)	1 (6.3)	15 (9.0)	78 (18.4)	26 (11.6)	4 (11.8)	9 (8.5)	13 (15.5)	25 (5.8)	1 (1.6)	16 (8.2)	8 (4.5)
OOPEs per mo												
Median (IQR), $	74.50 (10.00-309.00)	75.50 (74.50-149.50)	10.00 (0-74.50)	149.50 (74.50-278.00)	10.00 (0-74.50)	17.25 (0-278.00)	0 (0-10.00)	34.50 (34.50-149.50)	10.00 (0-74.50)	0 (0-34.50)	0 (0-10.00)	74.50 (34.50-149.50)
≥$50	329 (54.3)	12 (75.0)	41 (24.6)	276 (65.3)	60 (26.8)	14 (41.2)	12 (11.3)	34 (40.5)	114 (26.3)	14 (23.0)	15 (7.7)	85 (48.0)
≥$100	236 (38.9)	5 (31.3)	26 (15.6)	205 (48.5)	41 (18.3)	11 (32.4)	5 (4.7)	25 (29.8)	68 (15.7)	11 (18.0)	9 (4.6)	48 (27.1)

Abbreviations: OOPEs, out-of-pocket expenses; SEARCH, SEARCH for Diabetes in Youth; T1D, type 1 diabetes; T2D, type 2 diabetes; TODAY, Treatment Options for Type 2 Diabetes in Adolescents and Youth.

^a^ Care included care for diabetes, other medical problems, and dental health. The OOPEs included those for services that were not covered by health insurance plan, but not the purchase price of the health insurance plan. The mean OOPEs were calculated as the weighted mean based on the midpoint for each interval and the number of participants whose income falls within each interval. The midpoint of the last interval (≥$200) was estimated at $309 based on 2020 MarketScan data.

Participants with T1D were more likely than those with T2D in both SEARCH and TODAY studies to have 1 or more visits for diabetes care in the prior 6 months (88.1%, 80.5%, and 73.6%, respectively) (Table 3). Participants with T1D and no coverage were less likely to have at least 1 visit in the prior 6 months (62.5%) compared with those with public (90.4%) or private (87.5%) coverage. For those with T2D, there were no major differences in the number of diabetes care visits among those with and without health care coverage in either study.

Participants with T1D reported higher OOPEs for medical care compared with those with T2D. Median (IQR) OOPEs per month were $74.50 ($10.00-$309.00) for T1D and $10.00 ($0-$74.50) for the T2D group in both SEARCH and TODAY studies (Table 3). Additionally, 54.3% of participants with T1D compared with 26.8% of participants with T2D in the SEARCH study and 26.3% of participants with T2D in the TODAY study had OOPEs of at least $50 per month. Only 14.0% of participants with T1D reported no OOPEs compared with 38.0% of participants with T2D in the SEARCH study and 39.5% of participants with T2D in the TODAY study.

Reported OOPEs were inversely associated with health care coverage in participants with T1D. Those without health care coverage were more likely to report having OOPEs compared with those with health care coverage (81.3% vs 70.2%) (Table 3). The association between OOPEs and health care coverage was different in participants with T2D. Those with T2D and health care coverage in SEARCH and TODAY studies (51.6% and 57.5%) were more likely to report having OOPEs compared with those without health care coverage (44.1% and 37.7%).

In minimally adjusted models, not having health care coverage and not having an established source of diabetes care were associated with significantly higher mean (SE) HbA_1c_ levels in participants with T1D (no coverage: 10.8% [0.5%], public: 9.4% [0.2%], private: 8.7% [0.1%], *P* < .001; source of care, no: 10.7% [0.4%], yes: 8.9% [0.1%], *P* < .001); there were no associations for those with T2D in either study (Table 4). Adjusting for insulin pump use, frequency of glucose monitoring, and depressed mood for the T1D group and for diabetes medications and depressed mood for the T2D group did not change the results in the SEARCH study. However, in fully adjusted models, not having health care coverage and not having an established source of diabetes care were associated with significantly higher mean (SE) HbA_1c_ levels in TODAY T2D study participants (no coverage: 9.9% [0.3%], public: 8.7% [0.2%], private: 8.7% [0.2%], *P* = .004; source of care, no: 9.7% [0.2%], yes: 9.0% [0.2%], *P* = .01).

**Table 4.** Mean HbA_1c_ Levels by Health Care Coverage, Usual Source of Diabetes Care, and Frequency of Use by Linear Regression Models^a^

	Mean (SE) HbA_1c_ levels, %								
	SEARCH study cohort						TODAY T2D study cohort		
	T1D			T2D			Model 1	Model 2	Model 3
	Model 1	Model 2	Model 3	Model 1	Model 2	Model 3			
Health care coverage in past 12 mo	n = 592	n = 592	n = 592	n = 222	n = 222	n = 222	n = 433	n = 433	n = 433
No coverage	10.8 (0.5)	10.6 (0.6)	10.7 (0.6)	10.3 (0.5)	9.7 (0.5)	9.7 (0.5)	9.7 (0.4)	10.0 (0.3)	9.9 (0.3)
Public	9.4 (0.2)	9.5 (0.2)	9.6 (0.2)	9.1 (0.3)	8.4 (0.4)	8.3 (0.4)	9.4 (0.2)	8.8 (0.2)	8.7 (0.2)
Private	8.7 (0.1)	9.0 (0.1)	9.1 (0.1)	9.5 (0.3)	9.0 (0.3)	8.9 (0.4)	9.2 (0.2)	8.8 (0.2)	8.7 (0.2)
*P* value	<.001	.002	.003	.13	.05	.06	.47	.004	.004
Usual source of care for diabetes	n = 606	n = 606	n = 606	n = 224	n = 224	n = 224	n = 433	n = 433	n = 433
No	10.7 (0.4)	10.8 (0.4)	10.8 (0.4)	9.5 (0.4)	9.1 (0.5)	9.1 (0.5)	9.4 (0.3)	9.8 (0.2)	9.7 (0.2)
Yes	8.9 (0.1)	9.1 (0.1)	9.2 (0.1)	9.5 (0.3)	8.7 (0.3)	8.6 (0.3)	9.3 (0.2)	9.1 (0.1)	9.0 (0.2)
*P* value	<.001	<.001	<.001	.99	.36	.32	.82	.02	.01
Frequency of use in past 6 mo	n = 574	n = 574	n = 574	n = 175	n = 175	n = 175	n = 318	n = 318	n = 318
0	9.3 (0.2)	9.4 (0.3)	9.5 (0.3)	9.6 (0.5)	9.1 (0.5)	9.0 (0.5)	9.0 (0.3)	9.0 (0.3)	8.9 (0.3)
1-2	8.8 (0.1)	9.0 (0.1)	9.1 (0.1)	9.7 (0.3)	8.8 (0.3)	8.7 (0.4)	9.5 (0.2)	8.7 (0.2)	8.5 (0.2)
≥3	9.6 (0.3)	9.8 (0.3)	9.9 (0.3)	9.4 (0.5)	8.2 (0.5)	8.1 (0.5)	9.1 (0.4)	8.1 (0.4)	8.0 (0.4)
*P* value	.002	.007	.02	.86	.30	.32	.23	.15	.14

Abbreviations: HbA_1c_, glycated hemoglobin; SEARCH, SEARCH for Diabetes in Youth; T1D, type 1 diabetes; T2D, type 2 diabetes; TODAY, Treatment Options for Type 2 Diabetes in Adolescents and Youth.

^a^ Linear regression models were used to evaluate the association between HbA_1c_ levels and health care coverage, usual source of care, and health care use groups. Least square means (SEs) are presented. Model 1 was adjusted for age, diabetes duration, and educational level. Model 2 was further adjusted for insulin pump use for T1D or diabetes medications for T2D, and frequency of glucose monitoring for T1D. Model 3 was further adjusted for depressed mood.

Residing in a state with Medicaid expansion compared with a state without Medicaid expansion was associated with lower unadjusted mean (SE) HbA_1c_ levels for participants with T1D (8.8% [0.1%] vs 9.6% [0.2%]; *P* < .001] and participants with T2D in the SEARCH study (9.1% [0.2%] vs 10.3% [0.3%]; *P* = .002) (eTable 4 in Supplement 1). These differences remained significant after adjusting for insulin pump use, frequency of glucose monitoring, and depressed mood for the T1D group and for diabetes medications and depressed mood for the T2D group. In fully adjusted models, Medicaid expansion vs without expansion was associated with lower mean (SE) HbA_1c_ levels in participants with T1D (9.2% [0.1%] vs 9.7% [0.2%]; *P* = .02) and participants with T2D in the SEARCH study (8.4% [0.3%] vs 9.3% [0.4%]; *P* = .01) and in the TODAY study (8.7% [0.2%] vs 9.3% [0.2%]; *P* = .03).

## Discussion

In this cohort study, patterns of health care coverage, access to, and use of diabetes care differed between the T1D and T2D groups. Participants with T2D were more likely to report having no health care coverage, less likely to have an established source of diabetes care, and reported a lower frequency of use of diabetes care compared with those with T1D. Reported OOPEs were markedly higher in participants with T1D compared with those with T2D, which was likely associated with higher costs of care, including insulin, insulin pumps, and frequent fingerstick or continuous glucose monitoring among participants with T1D, although this finding may also reflect participants with T2D who were not filling prescriptions or seeking care. Lack of health care coverage and lack of an established source of diabetes care were associated with higher HbA_1c_ levels in participants with T1D and participants with T2D in the TODAY study. A similar pattern, although not statistically significant, was observed in participants with T2D in the SEARCH study.

Access to diabetes care is an important issue for all younger adults with diabetes, and this study identified areas of limited access among both T1D and T2D populations. However, less access (ie, no health care coverage and no usual source of diabetes care) was more frequently reported by participants with T2D in both SEARCH and TODAY studies. Economic and social determinants of health likely play a key role here. The sociodemographic characteristics of sex, race and ethnicity, educational level, and household income of participants with T1D were different from those of participants with T2D. Less access to and use of diabetes care in those with T2D was especially alarming given emerging evidence that T2D diagnosed in youth follows a more aggressive clinical course and that youth and young adults with T2D have a higher frequency of diabetes complications than those with T1D of similar duration.^15,21,22,23^

The reasons for not having an established source of diabetes care varied by diabetes type. Participants with T1D reported having no coverage, changing insurance plans, and high costs as reasons for having no source of diabetes care. For participants with T2D, the commonly cited reasons were having no coverage, not feeling a need for care, and not knowing where to get care. The finding that many participants with T2D believed they did not need diabetes care because they were never or rarely sick suggests a need for dramatically more effective education regarding the potential implication of youth-onset T2D for health outcomes, especially given the high mean HbA_1c_ levels in the study population.

Health care coverage availability has been a policy issue in the US for decades. In this study, participants with T1D or T2D diabetes residing in states with expanded Medicaid programs reported higher frequencies of health care coverage and lower mean HbA_1c_ levels than participants from states without Medicaid expansion. This finding is encouraging because it provides a potential opportunity for improving diabetes care. However, previous studies report that social and economic factors, beyond access to care, also influence the health outcomes in persons with diabetes.^24^ Further work examining health equity and social determinants of health for young adults with diabetes could provide additional insights into these dynamics.^25^

The association of lack of health care coverage and lack of usual source of diabetes care with higher HbA_1c_ levels in participants with T1D, but not in those with T2D, is notable. Fewer health care visits due to not having an established source of care or not having health care coverage and leading to less clinical oversight may account for higher HbA_1c_ levels in younger adults with T1D. The role of access to and use of diabetes care in glycemic control in younger adults with T2D is less clear.

These study results involve patterns that existed before the COVID-19 pandemic. The long-term implication of the pandemic for health care access and diabetes care in younger adults with diabetes is yet to be determined. An increase in non–employer-sponsored health care coverage, such as Medicaid expansion, and stability in employer-sponsored health care coverage were observed in 2021 to early 2022,^26^ but continued coverage may expire in some US locations. Overall use of diabetes care declined in younger adults due to care center closures and delayed care decisions.^27^ There is concern that decisions to delay or forego care may extend into the future. However, there is some indication that Medicaid expansion was associated with higher rates of employment and pursuit of higher education, particularly among younger adults with chronic health conditions, which may enhance the use of health care in the future.^28^

### Strengths and Limitations

A major strength of this study is the collaborative effort between SEARCH and TODAY, 2 large studies of youth-onset diabetes (eAppendix in Supplement 1). Through this collaboration, we were able to compare patterns of health care coverage, access to, and use of diabetes care between participants with T1D and participants with T2D from these studies. In general, the TODAY study results validated the SEARCH study findings in T2D.

The study also has limitations. First, the results suggest that living in a state without Medicaid expansion may be an added risk factor for elevated HbA_1c_ level, for any health care coverage status or type. However, these data did not allow us to distinguish whether HbA_1c_ levels had a causal association with Medicaid expansion status or might be associated with other risk factors that may be more common in states without Medicaid expansion (eg, poverty and proximity to university-affiliated medical centers). This question has important implications for public policy and will need to be addressed in future studies. Second, while measurement error in the survey responses may exist, the questions in the survey were modeled after the questions in the national Medical Expenditure Panel Survey and involved shortened periods of recall. Neither the TODAY nor the SEARCH study performed reviews of health care claims data to validate reported use. Participants in the SEARCH and TODAY studies had a mean duration of diabetes of 12 years and numerous study visits over that period. Thus, these individuals were committed to the research studies. The study sites were mainly at academic centers, and the racial and ethnic distribution was reflective of the general population in the regions in which the studies were conducted. However, the findings may not be generalizable to all younger adults with diabetes, to rural populations, to individuals receiving care from private practice or public health centers, or to other US regions.

## Conclusions

In this cohort study, lack of health care coverage and lack of an established source of diabetes care were associated with significantly higher HbA_1c_ levels for participants with T1D, but inconsistent differences were observed for participants with T2D, suggesting that additional barriers, including both social and economic factors, exist for the T2D group. Participants with either T1D or T2D were more likely to have lower HbA_1c_ levels if they resided in states with Medicaid expansion. Ensuring that processes and structures of care optimize access to diabetes care and health care coverage for younger adults with diabetes could play a role in lower HbA_1c_ levels and improved health outcomes in this high-risk population. Effective education on optimizing diabetes care in younger adults is essential, given the sizeable portion of those who reported not seeking care because they rarely or never get sick yet had high mean HbA_1c_ levels. There is an urgent need for additional strategies to improve outcomes for younger adults with diabetes, particularly T2D.

## Notes

Supplement 1. **eFigure.** Medicaid Expansion Status at the Time of Data Collection and Location of SEARCH and TODAY Clinical Sites **eTable 1.** Survey Questions **eTable 2.** Health Care Coverage by Expanded Medicaid Status **eTable 3.** Health Care Coverage by Age (<26 vs. ≥26 Years) **eTable 4.** Mean HbA1c Levels by Expanded Medicaid Status and Health Care Coverage by Linear Regression Models **eAppendix.** SEARCH/TODAY Study Group List

Supplement 2.
Data Sharing Statement
