# Supplementary material for: Diabetes Care Barriers, Use, and Health Outcomes in Younger Adults With Type 1 and Type 2 Diabetes
Source: JAMA Netw Open. 2023 May 5;6(5):e2312147. doi: 10.1001/jamanetworkopen.2023.12147 (PMC10163384; doi:10.1001/jamanetworkopen.2023.12147)
Supplement: Supplement 1. — eFigure. Medicaid Expansion Status at the Time of Data Collection and Location of SEARCH and TODAY Clinical Sites eTable 1. Survey Questions eTable 2. Health Care Coverage by Expanded Medicaid Status eTable 3. Health Care Coverage by Age (<26 vs. ≥26 Years) eTable 4. Mean HbA1c Levels by Expanded Medicaid Status and Health Care Coverage by Linear Regression Models eAppendix. SEARCH/TODAY Study Group List [file jamanetwopen-e2312147-s001.pdf]

Supplemental Online Content

Pihoker C, Braffett BH, Songer TJ, et al; Writing Committee for the SEARCH for Diabetes in Youth Study Group and the TODAY Study Group. Diabetes care barriers, use, and health outcomes in younger adults with type 1 and type 2 diabetes. *JAMA Netw Open*. 2023;6(5):e2312147. doi:10.1001/jamanetworkopen.2023.12147

eFigure. Medicaid Expansion Status at the Time of Data Collection and Location of SEARCH and TODAY Clinical Sites

eTable 1. Survey Questions

eTable 2. Health Care Coverage by Expanded Medicaid Status

eTable 3. Health Care Coverage by Age (<26 vs. ≥26 Years)

eTable 4. Mean HbA1c Levels by Expanded Medicaid Status and Health Care Coverage by Linear Regression Models

eAppendix. SEARCH/TODAY Study Group List

This supplemental material has been provided by the authors to give readers additional information about their work.

© 2023 Pihoker C et al. *JAMA Network Open*.

eFigure. Medicaid Expansion Status at the Time of Data Collection and Location of SEARCH and TODAY Clinical Sites

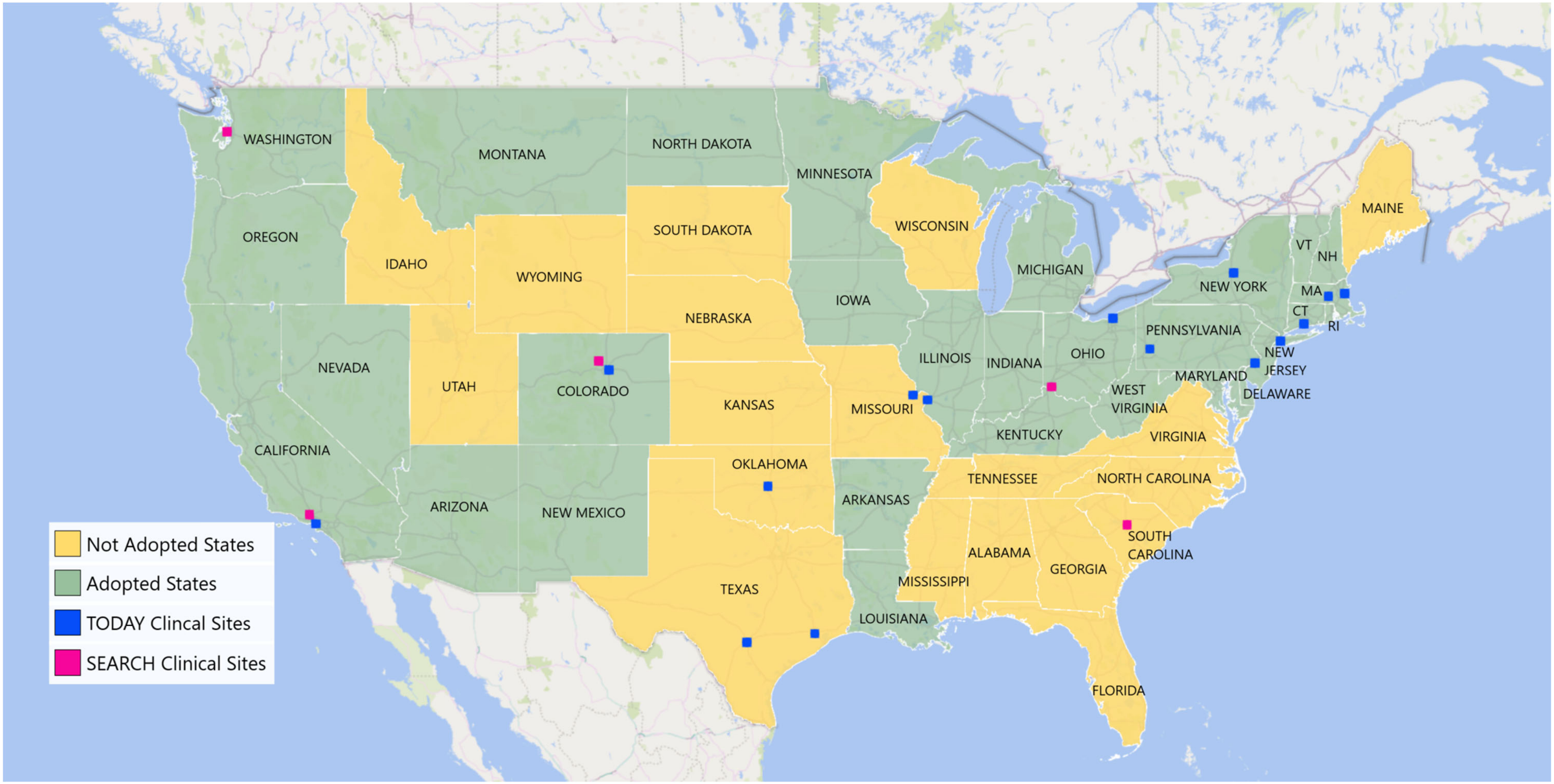

© 2023 Pihoker C et al. *JAMA Network Open*.

eTable 1. Survey Questions

Health Care Coverage

In the past 12 months, were you covered by a health care plan?

If Yes, who provided your health care plan?

Usual Source of Care

Is there a particular doctor's office, clinic health center, or other place where you usually go to get care for your diabetes?

If Yes, where?

If Yes, in the past 6 months, how often did you use this facility?

What are the reasons that you don't have a place where you usually go to get care for your diabetes? Of these, what is the main reason?

- Seldom or never get sick due to my diabetes
- Recently moved into the area
- Don't know where to go for care
- Usual source of medical care in this area is no longer available
- Can't find a provider who speaks my language
- Prefer to go to different places, not just one place
- Just changed insurance plans
- Don't use doctors or medical healthcare providers
- Can't afford medical care costs
- Have no health insurance
- Can't get off work or school
- Can't get to a location, no means of transportation
- Can't leave family members or others I have to care for
- Other reason
- Don't know ore refused to answer

Out-of-Pocket Health Care Expenses

On average what are your out-of-pocket expenses for health care per month? 'Health care includes care for your diabetes, for other medical problems, and for dental health. By 'out-of-pocket expenses' we mean costs for health care services not covered by insurance – but don't include what you send to purchase the insurance.

© 2023 Pihoker C et al. *JAMA Network Open*.

eTable 2. Health Care Coverage by Expanded Medicaid Status

Coverage	SEARCH study cohort, No. (%)				TODAY T2D study cohort, No. (%)	
	T1D		T2D			
	Not Expanded	Expanded	Not Expanded	Expanded	Not Expanded	Expanded
**Health care coverage status in past 12 mo, all participants**	**133 (100)**	**528 (100)**	**92 (100)**	**158 (100)**	**163 (100)**	**297 (100)**
No	12 (9.0)	4 (0.8)	24 (26.1)	10 (6.3)	42 (25.8)	19 (6.4)
Yes	120 (90.2)	506 (95.8)	68 (73.9)	136 (86.1)	121 (74.2)	278 (93.6)
Don’t know, refused to answer, or missing data	1 (0.8)	18 (3.4)	0 (0.0)	12 (7.6)	0 (0.0)	0 (0.0)
**Health care coverage type, all participants**	**120 (90.2)**	**506 (95.8)**	**68 (73.9)**	**136 (86.1)**	**121 (74.2)**	**278 (93.6)**
Public						
Medicaid, Medicare, CHIP, or other state or federal	28 (23.3)	132 (26.1)	31 (45.6)	72 (52.9)	38 (31.4)	144 (51.8)
Military (eg, TRICARE, CHAMPUS)	4 (3.3)	3 (0.6)	1 (1.5)	1 (0.7)	0 (0.0)	0 (0.0)
Indian Health Service or Tribal Nations	0 (0.0)	0 (0.0)	0 (0.0)	1 (0.7)	13 (10.7)	0 (0.0)
Private through work, parent or caregiver, or purchased individually	77 (64.2)	346 (68.4)	29 (42.7)	55 (40.4)	60 (49.6)	117 (42.1)
Mixed public and private	7 (5.8)	18 (3.6)	5 (7.4)	3 (2.2)	9 (7.4)	11 (4.0)
Other type or unknown	4 (3.3)	7 (1.4)	2 (2.9)	4 (2.9)	1 (0.8)	6 (2.2)
**Health care coverage status and type, collapsed sample[a]**	**121 (91.0)**	**485 (91.9)**	**85 (92.4)**	**139 (88.0)**	**153 (93.9)**	**280 (94.3)**
No coverage	12 (9.9)	4 (0.8)	24 (28.2)	10 (7.2)	42 (27.5)	19 (6.8)
Public	32 (26.5)	135 (27.8)	32 (37.7)	74 (53.2)	51 (33.3)	144 (51.4)
Private	77 (63.6)	346 (71.3)	29 (34.1)	55 (39.6)	60 (39.2)	117 (41.8)

[a] The collapsed sample excluded n=31 SEARCH participants who did not know, refused to answer, or were missing health care status; 33 SEARCH and 20 TODAY study participants with mixed public and private coverage; and 17 SEARCH and 7 TODAY participants with other or unknown health plan types.

© 2023 Pihoker C et al. *JAMA Network Open*.

eTable 3. Health Care Coverage by Age (<26 vs. ≥26 Years)

Coverage	SEARCH study cohort, No. (%)				TODAY T2D study cohort, No. (%)	
	T1D		T2D			
	<26 years	≥26 years	<26 years	≥26 years	<26 years	≥26 years
**Health care coverage status in past 12 mo, all participants**	**501 (100)**	**160 (100)**	**168 (100)**	**82 (100)**	**220 (100)**	**240 (100)**
No	12 (2.4)	4 (2.5)	18 (10.7)	16 (19.5)	30 (13.6)	31 (12.9)
Yes	474 (94.6)	152 (95.0)	141 (83.9)	63 (76.8)	190 (86.4)	209 (87.1)
Don't know, refused to answer, or missing data	15 (3.0)	4 (2.5)	9 (5.4)	3 (3.7)	0 (0.0)	0 (0.0)
**Health care coverage type, all participants**	**474 (94.6)**	**152 (95.0)**	**141 (83.9)**	**63 (76.8)**	**190 (86.4)**	**209 (87.1)**
Public						
Medicaid, Medicare, CHIP, or other state or federal	115 (24.3)	45 (29.6)	71 (50.4)	32 (50.8)	93 (49.0)	89 (42.6)
Military (eg, TRICARE, CHAMPUS)	6 (1.3)	1 (0.7)	1 (0.7)	1 (1.6)	0 (0.0)	0 (0.0)
Indian Health Service or Tribal Nations	0 (0.0)	0 (0.0)	0 (0.0)	1 (1.6)	6 (3.2)	7 (3.4)
Private through work, parent or caregiver, or purchased individually	323 (68.1)	100 (65.8)	60 (42.6)	24 (38.1)	80 (42.1)	97 (46.4)
Mixed public and private	21 (4.4)	4 (2.6)	5 (3.6)	3 (4.8)	7 (3.7)	13 (6.2)
Other type or unknown	9 (1.9)	2 (1.3)	4 (2.8)	2 (3.2)	4 (2.1)	3 (1.4)
**Health care coverage status and type, collapsed sample[a]**	**456 (91.0)**	**150 (93.8)**	**150 (89.3)**	**74 (90.2)**	**209 (95.0)**	**224 (93.3)**
No coverage	12 (2.6)	4 (2.7)	18 (12.0)	16 (21.6)	30 (14.4)	31 (13.8)
Public	121 (26.5)	46 (30.7)	72 (48.0)	34 (46.0)	99 (47.4)	96 (42.9)
Private	323 (70.8)	100 (66.7)	60 (40.0)	24 (32.4)	80 (38.3)	97 (43.3)

[a] The collapsed sample excluded n=31 SEARCH participants who did not know, refused to answer, or were missing health care status; 33 SEARCH and 20 TODAY study participants with mixed public and private coverage; and 17 SEARCH and 7 TODAY participants with other or unknown health plan types.

© 2023 Pihoker C et al. *JAMA Network Open*.

eTable 4. Mean HbA1c Levels by Expanded Medicaid Status and Health Care Coverage by Linear Regression Models[a]

	SEARCH study cohort								TODAY T2D study cohort			
	T1D				T2D							
	Model 1		Model 2		Model 1		Model 2		Model 1		Model 2	
	Not Expand	Expand	Not Expand	Expand	Not Expand	Expand	Not Expand	Expand	Not Expand	Expand	Not Expand	Expand
**All participants**	**n=133**	**n=528**	**n=133**	**n=528**	**n=92**	**n=158**	**n=92**	**n=158**	**n=163**	**n=297**	**n=163**	**n=297**
	9.6 (0.2)	8.8 (0.1)	9.7 (0.2)	9.2 (0.1)	10.3 (0.3)	9.1 (0.2)	9.3 (0.4)	8.4 (0.3)	9.5 (0.2)	9.2 (0.2)	9.3 (0.2)	8.7 (0.2)
P value	<.001		.02		.002		.01		.27		.03	
**Health care coverage in past 12 mo**	**n=120**	**n=472**	**n=120**	**n=472**	**n=84**	**n=138**	**n=84**	**n=138**	**n=153**	**n=280**	**n=153**	**n=280**
No coverage	11.4 (0.7)	9.9 (1.0)	11.2 (0.8)	8.9 (1.0)	10.6 (0.6)	9.6 (0.9)	9.6 (0.7)	10.4 (0.9)	10.1 (0.4)	9.2 (0.6)	10.1 (0.4)	9.5 (0.6)
Public	9.8 (0.4)	9.5 (0.2)	9.8 (0.5)	9.5 (0.2)	9.8 (0.5)	9.0 (0.3)	8.7 (0.6)	8.2 (0.5)	9.3 (0.4)	9.5 (0.2)	8.9 (0.4)	8.7 (0.2)
Private	9.0 (0.3)	8.5 (0.1)	9.3 (0.3)	9.1 (0.1)	10.6 (0.5)	9.0 (0.4)	9.6 (0.6)	8.5 (0.4)	9.4 (0.4)	8.9 (0.3)	9.1 (0.4)	8.6 (0.2)
P value	.005	<.001	.09	.08	.43	.77	.45	.10	.32	.30	.08	.34

[a] Linear regression models were used to evaluate the association between HbA1c levels and health care coverage. Least squares means (SEs) are presented. Model 1 was unadjusted. Model 2 was adjusted for age, diabetes duration, educational level, insulin pump use for T1D or diabetes medications for T2D, frequency of glucose monitoring for T1D, and depressed mood.

© 2023 Pihoker C et al. *JAMA Network Open*.

eAppendix. SEARCH/TODAY Study Group List

The writing group for this manuscript wishes to acknowledge the contributions of the following individuals to the SEARCH for Diabetes in Youth and Treatment Options for type 2 Diabetes in Adolescents and Youth (TODAY) studies.

SEARCH STUDY GROUP LIST

SEARCH SITES

California: Jean M. Lawrence, ScD, MPH, MSSA*
Peggy Hung, MPH; Corinna Koebnick, PhD, MSc; Xia Li, MS; Eva Lustigova, MPH; Kristi Reynolds, PhD, MPH for the Department of Research & Evaluation, Kaiser Permanente Southern California, Pasadena California, and David J. Pettitt, MD, Santa Barbara, California. *Dr. Lawrence is now with the National Institute of Diabetes and Digestive and Kidney Diseases.

Carolinas: Elizabeth J. Mayer-Davis, PhD*
Amy Mottl, MD, MPH; Joan Thomas MS, RD for the University of North Carolina, Chapel Hill.

Malaka Jackson, MD; Lisa Knight, MD; Angela D. Liese, PhD, MPH; Christine Turley, MD for the University of South Carolina.

Deborah Bowlby, MD for the Medical University of South Carolina.

James Amrhein, MD; Elaine Apperson, MD; Bryce Nelson, MD for Greenville Health System and Eau Claire Cooperative Health Center.

Colorado: Dana Dabelea, MD, PhD*
Anna Bellatorre, PhD; Tessa Crume, PhD, MSPH; Richard F. Hamman, MD, DrPH; Katherine A. Sauder, PhD; Allison Shapiro, PhD, MPH; Lisa Testaverde, MS for the LEAD Center in the Department of Epidemiology, Colorado School of Public Health, University of Colorado Denver.

Georgeanna J. Klingensmith, MD; David Maahs, MD; Marian J. Rewers, MD, PhD; Paul Wadwa, MD for the Barbara Davis Center for Childhood Diabetes.

Stephen Daniels, MD, PhD; Michael G. Kahn, MD, PhD; Greta Wilkening, PsyD for the Department of Pediatrics and Children's Hospital.

Clifford A. Bloch, MD for the Pediatric Endocrine Associates.

Jeffrey Powell, MD, MPH for the Shiprock Service Unit, Navajo Area Indian Health Service.

Kathy Love-Osborne, MD for the Denver Health and Hospital Authority.

Diana C. Hu, MD for the Pediatrics Department, Tuba City Regional Health Care Center, Tuba City, AZ.

Ohio: Lawrence M. Dolan, MD*
Amy S. Shah, MD, MS; Debra A. Standiford, MSN, CNP; Elaine M. Urbina, MD, MS for the Cincinnati Children's Hospital Medical Center, Department of Pediatrics, University of Cincinnati.

Washington: Catherine Pihoker, MD*
Irl Hirsch, MD; Grace Kim, MD; Faisal Malik, MD, MSHS; Lina Merjaneh, MD; Alissa Roberts, MD; Craig Taplin, MD; Joyce Yi-Frazier, PhD for the University of Washington.

Natalie Beauregard, BA; Cordelia Franklin, BS; Carlo Gangan, BA; Sue Kearns, RN; Mary Klingsheim, RN; Beth Loots, MPH, MSW; Michael Pascual, BA for Seattle Children's Hospital.

Carla Greenbaum, MD for Benaroya Research Institute.

FUNDING AGENCIES, COORDINATING CENTER and LABORATORY

Centers for Disease Control and Prevention: Giuseppina Imperatore, MD, PhD, Sharon H. Saydah, PhD

National Institute of Diabetes and Digestive and Kidney Diseases, NIH: Barbara Linder, MD, PhD

Central Laboratory: Santica M. Marcovina, PhD, ScD*
Alan Chait, MD; Noemie Clouet-Foraison, PhD; Jessica Harting; Greg Strylewicz, PhD for the University of Washington Northwest Lipid Metabolism and Diabetes Research Laboratories.

Coordinating Center: Ralph D'Agostino, Jr., PhD*, Elizabeth T. Jensen, MPH, PhD*; Lynne E. Wagenknecht, DrPH; Ronny A. Bell, PhD; Ramon Casanova, PhD; Jasmin Divers, PhD; Maureen T. Goldstein, BA; Leora Henkin, MPH, M.Ed; Scott Isom, MS; Kristin Lenoir, MPH; June Pierce, AB; Beth Reboussin, PhD; Joseph Rigdon, PhD; Andrew Michael South, MD, MS; Jeanette Stafford, MS; Cynthia Suerken, MS; Brian Wells, MD, PhD; Carrie Williams, MA, CCRP for Wake Forest School of Medicine.

© 2023 Pihoker C et al. *JAMA Network Open*.

TODAY STUDY GROUP LIST

CLINICAL CENTERS

Baylor College of Medicine: S. McKay*, B. Anderson, F. Bacha, S. Gunn, M. Haymond, N. Miranda, S. Seributra, R. Zagado

Case Western Reserve University: R. Gubitosi-Klug* (Steering Committee Vice-Chair), S. Narasimhan*, T. Casey, R. Farrell, K. Kutney, S. Macleish, P. McGuigan

Children's Hospital Los Angeles: M. Geffner*, N. Chang, L. Chao, J. Quach, L. Fisher, V. Guzman

Children's Hospital of Philadelphia: L. Levitt Katz*, C. Carchidi, G. McGinley, B. Schwartzman, R. Shah, S. Willi

Children's Hospital of Pittsburgh: S. Arslanian*, K. Brown, S. Cochenour, A. Flint, N. Guerra, K. Hughan, I. Libman, M. Marcus, K. Porter

Columbia University Medical Center: R. Goland*, R. Gandica, K. Gumpel, C. Hausheer, P. Kringas, J. Pring

Joslin Diabetes Center: L. Laffel*, L. Higgins, E. Isganaitis, J. Keady, M. Malloy, K. Milaszewski

Massachusetts General Hospital: D.M. Nathan*, D. Koren, L. Levitsky, K. Martin, B. Steiner

Saint Louis University: S. Tollefsen*, T. Cattoor, D. Dempsher, J. Meyer, K. Schopp, M. Siska, B. Wolff

State University of New York Upstate Medical University: R. Weinstock*, S. Bzdick, P. Conboy

University of Colorado Denver: P. Zeitler* (Steering Committee Chair), N. Abramson, P. Bjornstad, C. Chan, J. Higgins, C. Hovater, M.M. Kelsey, K. Nadeau, C. Retamal-Munoz, K. Vissat

Oklahoma Health Sciences Center: J. Tryggestad*, S. Chernausek*, K. Copeland* (retired), J. Chadwick, J. Less, C. Macha, J. Preske

University of Texas Health Science Center at San Antonio: J. Lynch*, R. Barajas, E. Escaname, D. Hale, C. Orsi, M. Rayas, A. Wauters, D. Word

Washington University in St Louis: N. White*, J. Jones, T. Jones, T. Stich

Yale University: S. Caprio*, C. Guandalini, P. Rose, M. Van Name

COORDINATING CENTER

George Washington University Biostatistics Center: K. Drews*, B. Braffett, B. Burke, K. Cross, L. El ghormli, J. George, N. Grover, M. Gunaratne, A. Lauer, K. Tan, B. Tesfaldet, M. Tung, M. Turney, D. Uschner, S. Zhou

PROJECT OFFICE

National Institute of Diabetes and Digestive and Kidney Diseases: B. Linder*

© 2023 Pihoker C et al. *JAMA Network Open*.

CENTRAL UNITS

Central Blood Laboratory (Northwest Lipid Research Laboratories, University of Washington): S.M. Marcovina*, J. Albers, J. Harting, P. Parbhakar, J. Ramirez, M. Ramirez, G. Strylewicz

Echocardiogram Reading Center (Johns Hopkins University): J. Lima*, H. Doria de Vasconellos, S Gidding, K. Keck, J. Ortman, J. Puccella

Fundus Photography Reading Center (University of Wisconsin): B. Blodi*, M. Mititelu*, A. Domalpally

Pulse Wave Velocity Reading Center (Cincinnati Children's Hospital Medical Center): E. Urbina*, A. Shah

Sleep Reading Center (University of Chicago): B. Mokhlesi*, H. Whitmore

* Principal Investigator

© 2023 Pihoker C et al. *JAMA Network Open*.
