# Supplementary material for: Diabetes Care Barriers, Use, and Health Outcomes in Younger Adults With Type 1 and Type 2 Diabetes
Source: JAMA Netw Open. 2023 May 5;6(5):e2312147. doi: 10.1001/jamanetworkopen.2023.12147 (PMC10163384; doi:10.1001/jamanetworkopen.2023.12147)
Supplement: Supplement 2. — Data Sharing Statement [file jamanetwopen-e2312147-s002.pdf]

Data Sharing Statement

Pihoker. Diabetes Care Barriers, Use, and Health Outcomes in Younger Adults With Type 1 and Type 2 Diabetes. *JAMA Netw Open*. Published May 05, 2023.
doi:10.1001/jamanetworkopen.2023.12147

Data
Data available: Yes
Data types: Deidentified participant data
How to access data: Anonymized data and materials from the SEARCH and TODAY studies have been made publicly available at the National Institute of Diabetes and Digestive and Kidney Disease (NIDDK) Central Repository (https://repository.niddk.nih.gov/studies/search/ and https://repository.niddk.nih.gov/studies/today/).
When available: With publication

Supporting Documents
Document types: None

Additional Information
Who can access the data: Researchers whose proposed use of the data has been approved by the NIDDK Central Repository.
Types of analyses: As approved by the NIDDK Central Repository.
Mechanisms of data availability: As approved by the NIDDK Central Repository.
